# Comparing Levels of Metabolic Predictors of Coronary Heart Disease between Healthy Lean and Overweight Females

**DOI:** 10.3390/metabo11030169

**Published:** 2021-03-15

**Authors:** Rasha Abu-El-Ruz, Manar E. Abdel-Rahman, Stephen L. Atkin, Mohamed A. Elrayess

**Affiliations:** 1 Department of Public Health at the College of Health Sciences, QU Health, Qatar University, P. O. Box 2713 Doha, Qatar; rasha@qu.edu.qa (R.A.-E.-R.); melhassan@qu.edu.qa (M.E.A.-R.); 2 Research, Royal College of Surgeons in Ireland Bahrain, P. O. Box 15503 Adliya, Bahrain; satkin@rcsi-mub.com; 3 Biomedical Research Center, Qatar University, P. O. Box 2713 Doha, Qatar

**Keywords:** metabolomics, coronary heart disease, biomarkers, overweight

## Abstract

Screening for the metabolomic signature of coronary heart disease (CHD) before disease onset could help in early diagnosis and potentially disease prevention. In this study, the levels of 17 CHD metabolic biomarkers in apparently healthy overweight females were compared to lean counterparts, and their associations with conventional clinical risk factors were determined. Clinical and metabolic data from 200 apparently healthy non-obese Qatari females were collected from Qatar Biobank (discovery cohort). Logistic regression was used to assess the association between body mass index (BMI) groups and 17 CHD metabolic biomarkers, and receiver operating characteristic (ROC) analysis was used to evaluate the prognostic value of CHD metabolic biomarkers in overweight. Stepwise linear regression was performed to identify the classical risk factors associated with CHD metabolites differentiating the two BMI groups. Validation of the association of CHD metabolic biomarkers with BMI groups was performed in 107 subjects (replication cohort). Out of the tested CHD metabolic biomarkers, five were significantly different between lean and overweight females in the discovery cohort (AUC = 0.73). Among these, the association of mannose, asparagine, and linoleate with BMI groups was confirmed in the replication cohort (AUC = 0.97). Significant correlations between predictors of CHD in overweight healthy women and classical risk factors were observed, including serum levels of cholesterol, testosterone, triiodothyronine, thyroxine, creatinine, albumin, bilirubin, glucose, c-peptide, uric acid, calcium and chloride. Apparently, healthy overweight females exhibit significantly different levels of specific CHD metabolites compared to their lean counterparts, offering a prognostic potential with preventative value.

## 1. Introduction

Coronary heart disease (CHD) remains the prime cause of death worldwide [1]. Obesity has long been associated with an increased risk of cardiovascular morbidity and mortality compared with normal weight [2]. Obesity-associated cardiovascular complications are influenced by inflammation, insulin resistance, endothelial dysfunction, coronary calcification, activation of coagulation, renin-angiotensin, or the sympathetic nervous systems [3]. Overweight also has been associated with a significantly increased risk of developing CHD at an earlier age, resulting in a greater CHD morbidity, despite exhibiting similar longevity compared with normal body mass index (BMI) [4]. Interventions to reduce weight before the onset of obesity could significantly influence the development of risk factors and cardiovascular disease at individual and population levels [5].

The identification of apparently healthy individuals at higher risk of CHD before disease onset offers a window of opportunity to delay or prevent disease onset. The integration of classical risk factors with novel biomarkers could significantly help in assessing CHD risk [6]. Metabolomics is one of the most recent disciplines that describe the association of metabolites to diseases [7]. The application of metabolomics can provide an added value in various therapeutic and preventative measures for many chronic diseases such as heart diseases, cancer and diabetes [8,9,10,11,12]. Metabolic profiling of blood and body secretions could provide powerful diagnostic and potentially therapeutic tools [13]. Chronic diseases, such as cardiovascular disease, involve metabolomic changes that contribute to enhancing or worsening cellular functions [14].

Metabolic profiling of 3598 African and European Americans with 30 years follow-up has identified a set of 19 metabolites, including amino acids, lipids, peptides, carbohydrates, nucleotide, and xenobiotics, which were collectively associated with CHD risk [15]. However, the utility of these metabolic signatures in determining the risk of CHD in apparently healthy overweight females compared to age and sex-matched lean counterparts remains to be investigated. In this study, the primary objectives were to compare the levels of the metabolic signatures of CHD between apparently healthy lean and overweight females and to assess the accuracy and sensitivity of a classifier based on the identified metabolites to differentiate between the two groups in comparison to a classifier based on classical risk factors. The study also aims to identify correlations between predictors of CHD in overweight healthy women and classical risk factors.

## 2. Results

### 2.1. General Characteristics of Participants

Two hundred young (29.1 ± 5.4 years) non-obese (24.7 ± 2.8 kg/m^2^) females were classified into lean (body mass index, BMI < 25, *n* = 108) and overweight (BMI ≥ 25 and <30, *n* = 92) groups based on BMI index. The overweight women had significantly higher uric acid, total cholesterol, LDL-cholesterol, insulin and HOMA-IR compared to age-matched lean counterparts (Table 1).

### 2.2. Classical CHD Traits Associated with Overweight

**Figure 1:**
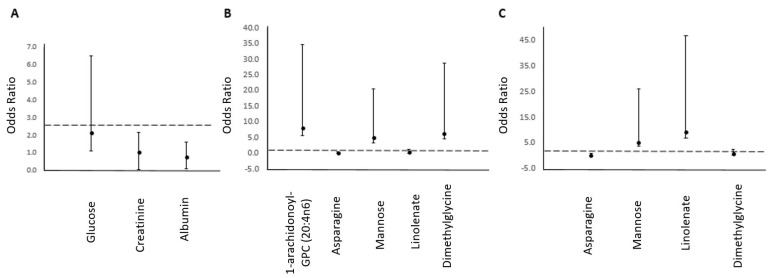
Individual metabolite odds ratios (95% CI) for (**A**) three classical traits (glucose, creatinine and albumin), (**B**) five CHD metabolites in the discovery cohort (1-arachidoniolyl 1-arachidonoyl-glycerylphosphorylcholine (GPC), asparagine, mannose, linolenate and dimethylglycine) and (**C**) four CHD metabolites in replication cohort (asparagine, mannose, linolenate and dimethylglycine).

**Figure 2:**
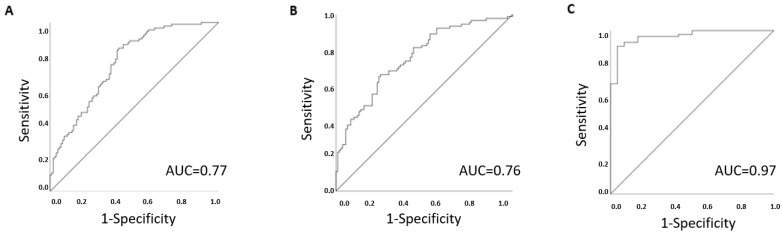
Receiver operating characteristic (ROC) curve indicates best predictors of BMI groups using (**A**) three classical traits (glucose, creatinine and albumin), (**B**) five CHD metabolites in the discovery cohort (1-arachidoniolyl GPC, asparagine, mannose, linolenate and dimethylglycine) and (**C**) four CHD metabolites in replication cohort (asparagine, mannose, linolenate and dimethylglycine). Area under the curve (AUC) for each model indicates the prognostic value of the classifier.

Out of the classical CHD risk factors listed in Table 1, a multivariable linear regression analysis revealed significant changes in three traits (creatinine, albumin and glucose) between lean and overweight participants (Table 2, Figure 1A). Accordingly, a ROC analysis has revealed that a classifier based on the identified traits can differentiate the two groups with an area under the curve (AUC) of 0.77 [95% CI: 0.6−0.8] (Table 2 and Figure 2A).

### 2.3. CHD Metabolites Associated with Being Overweight

Out of the 19 previously determined CHD metabolites, only 17 were detected in our samples (Table 3). Accordingly, multivariable linear regression analysis revealed significant changes in five of the 17 detected CHD metabolites between the two BMI groups (Table 2). These include two amino acids (asparagine and dimethylglycine), two lipids (1-arachidonoyl-GPC (20:4n6) and linolenate alpha or gamma; (18:3n3 or 6)) and one carbohydrate (mannose). Mannose, dimethyl-glycine and 1-arachidonoyl-GPC (20:4n6) were significantly higher in the overweight group, whereas levels of asparagine and linolenate were elevated in the lean group (Table 3 and Figure 1B). Adjustment for creatinine as a potential confounder did not alter the significance level of changes in metabolites levels with the BMI groups (data not shown). Accordingly, a ROC analysis has revealed that a classifier based on the identified metabolites can differentiate the two groups with an AUC of 73% (Figure 2B).

### 2.4. Validation of the Identified CHD Biomarkers Differentiating BMI Groups in an Independent Cohort

Validation of four available CHD metabolites (1-arachidonoyl-GPC was not available) was performed in a cohort of 107 subjects (BMI < 25, *n* = 24, and BMI ≥ 25, *n* = 83). The results indicated that three metabolites (mannose, asparagine and linolenate) were significantly different between the BMI groups (Table 2, Figure 1C), providing independent validation of associations identified in the discovery cohort in a second independent cohort. A ROC analysis has revealed that a classifier based on the identified traits can differentiate the two groups with an AUC of 0.97 (95% CI: 0.9–1.0) (Table 2 and Figure 2C), whereas a ROC analysis based on the classical risk traits (creatinine, albumin and glucose) exhibited an AUC of 0.76 (95% CI: 0.59–0.94). Interestingly, when comparing lean vs. obese samples using the same predictive metabolites (BMI ≥ 30, *n* = 75), the model remains predictive with an AUC of 0.99 (95% CI: 0.97–1.0).

### 2.5. Classical Risk Factors Associated with CHD Metabolites Differentiating Lean from Overweight Females

A stepwise linear regression analysis revealed the classical risk factors associated with confirmed CHD metabolites differentiating the two studied BMI groups (Table 4). These included lower total cholesterol and total testosterone but higher sex-hormone-binding globulin and calcium in association with asparagine. For predictors of mannose, classical risk factors included higher sex hormone-binding globulin, glucose, BMI, total bilirubin. For 1-arachidonoyl-GPC (20:4n6), predictors included elevated total cholesterol, alkaline phosphatase and free thyroxine, but lower homocysteine, LDL-cholesterol, total testosterone and chloride. Finally, predictors of linolenate (alpha or gamma; (18:3n3 or 6)) included elevated c-peptide, DBP, free triiodothyronine and total iron-binding capacity.

## 3. Discussion

Alterations in the serum metabolome can be detectable in individuals at risk of CHD before the onset of disease [15]. However, whether these CHD-associated metabolites can differentiate overweight individuals from lean counterparts remains unknown. The aim of this study was not to identify novel CHD biomarkers but to validate the existing ones in non-obese individuals and to investigate whether they can differentiate between lean (low-risk) and overweight (higher risk) groups of apparently healthy females. The ultimate goal of this study is to provide evidence for the use of these metabolites as potential predictive biomarkers of increased CHD risk in apparently healthy groups of individuals in future prospective studies.

In the discovery cross-sectional study containing census data from young non-obese healthy females, we assessed whether CHD metabolites could differentiate BMI groups. Our data indicated that five metabolites out of 17 detected CHD metabolites were indeed significantly different between the overweight and lean apparently healthy females. Our data also indicated that top classical CHD risk factors, including glucose, creatinine and albumin, showed significant differences with the BMI group. ROC analyses indicated a comparable prognostic value of the two models (CHD metabolites vs. classical risk factors) in differentiating BMI groups. Validation of four of these metabolites in the replication cohort confirmed the association of three metabolites (asparagine, mannose, and linolenate), but not the fourth (dimethylglycine), with BMI groups. Data from the fifth metabolite 1-arachidonoyl-GPC (20:4n6) was not available in the replication cohort. ROC analysis using confirmed metabolites in the replication cohort has confirmed the prognostic value of the model in differentiating BMI groups in a manner superior to that produced using the classical risk factors. Our data also identified changes in classical risk factors that are associated with CHD metabolites. These included alterations in the serum levels of lipids (total cholesterol and LDL), hormones (testosterone, T3 and T4), minerals (calcium and chloride) and proteins (bilirubin), among others.

In line with our data, previous studies have identified metabolites involved in amino acid metabolism, such as asparagine, to be associated with CHD occurrence [15,16,17,18,19]. However, our data reveal the predictive value of these metabolites in overweight females compared to age-matched lean counterparts. Asparagine, a derivative of aspartic acid, plays a critical role in glycoproteins biosynthesis [20]. It serves as a nontoxic carrier of residual ammonia for elimination. Although it was not confirmed in the replication cohort, dimethylglycine, a derivative of the amino acid glycine with a role in immune response [21], was previously used in prediction models of diabetes and CHD [22]. Studies have also shown a negative association of dimethylglycine with the risk of secondary heart failure, acute myocardial infarction and risk of CHD [23].

Other CHD metabolites that significantly changed between BMI groups were the lipids, including alpha-linolenic acid and 1-arachidonoylglycerophosphocholine (20:4n6). Alpha-linoleic acid, an omega-3 essential fatty acid, has a cardioprotective role [24]. It was previously identified to be negatively associated with CHD risk, which is in line with previous findings suggesting that polyunsaturated fatty acids are inversely associated with cardiovascular disease [25,26]. The other lipid metabolite, 1-arachidonoylglycerophosphocholine (20:4n6) (LysoPC 20:4), was not confirmed in the replication cohort, despite previous reports indicating its negative association with CHD [15]. LysoPC is mainly formed by the enzyme lecithin: cholesterol acyltransferase, which converts cholesterol and phosphatidylcholine to cholesterol esters and lysophosphatidylcholine [27,28]. Lower LysoPC 20:4 levels were reported in heart failure patients with reduced ejection fraction as compared to controls [29]. LysoPC (20:4) has been suggested to be a potential biomarker for discriminating CHD patients from controls (lower levels in CHD patients) [30].

The last CHD metabolite differentiating the BMI groups was mannose that plays an important role in glycolysis and gluconeogenesis. Previous reports suggested an association between elevated levels of mannose and the risk of CHD [31]. Studies indicated that increased plasma levels of mannose are associated with an elevated risk of CHD over time [32,33]. Our novel data confirm the potential predictive of these CHD metabolites in overweight.

In order to identify the classical risk factors associated with CHD metabolites differentiating the two studied groups, a stepwise linear regression revealed significant associations between these metabolites and various classical mediators of metabolic syndrome. These included association between asparagine and lower cholesterol and testosterone levels, but higher concentrations of sex hormone-binding globulin and calcium. The lower level of asparagine, shown previously to precede coronary artery disease [34], in the high BMI group is associated with higher concentrations of cholesterol and testosterone. Elevated levels of cholesterol and testosterone were previously shown to be associated with increased CHD risk in postmenopausal women [35], perhaps contributing to the increased risk associated with lower asparagine. Elevated levels of mannose, previously shown to be associated with increased CHD risk [15], was accompanied by higher levels of sex hormone-binding globulin, glucose, total bilirubin, all shown previously to be increased with a higher risk of CHD [36,37,38]. Elevation in these established risk factors could too partially explain increased CHD risk associated with higher levels of mannose in the high BMI group. The elevated level of 1-arachidonoyl-GPC (20:4n6), shown previously to be linked with increased CHD risk [15], was associated with increased cholesterol, alkaline phosphatase and T4, but lower homocysteine, LDL-cholesterol, total cholesterol and chloride. Elevated serum alkaline phosphatase and T4 were shown previously to be associated with the risk of CHD [39,40], possibly contributing to the increased CHD risk associated with the higher BMI group. Linolenate (18:3n3 or 6) was associated with elevated c-peptide, DBP, T3 and total iron-binding capacity. Elevated levels of linolenate were shown to be associated with increased CHD risk [15]. In addition to the established association between DBP and T3 with CHD risk, elevated c-peptide levels are associated with increased risk of CHD [41], perhaps contributing to increased CHD risk in the higher BMI group. Whether these associations are mediating the increased CHD risk in the overweight group or merely acting as a consequence of overweight-associated CHD risk, it remains to be investigated.

Although other studies have reported metabolic biomarkers of CHD in general, this is the first study to evaluate whether a selected set of CHD metabolites could differentiate BMI groups and potentially predict increased risk of CHD in apparently healthy overweight females. The significance of this finding once validated in other prospective cohorts based on CHD incidents is the potential to predict the risk of CHD in this healthy group of individuals based on a limited number of metabolites. However, the study suffers from certain limitations, including small sample size and a lack of longitudinal metabolomics data to validate the predictive power of these metabolites in relation to CHD risk. The study also suffers from the limited availability of classical biomarkers of CHD risk such as lipids, blood pressure, blood sugar and hormones. The study could have benefited significantly from adding other more relevant classical predictors of heart disease risk, such as data from an electrocardiogram, vascular scan, computerized tomography scan, cardiac magnetic resonance imaging, echocardiogram, coronary angiography, or cardiac biomarkers such as troponin and creatinine kinase. Furthermore, the data are limited to one ethnic group and gender; therefore, confirmation in other ethnicities and in males is warranted. Another limitation is related to the focused metabolic panel on small molecules but not complex lipids. Information on complex lipids, achieved through comprehensive lipidomic profiling, may provide additional insights for CHD risk [26,42]. Future replication in prospective studies is warranted to validate these findings in other independent populations to examine the value of metabolic changes in relation to CHD risk prediction, cost and feasibility of measurement assays and evidence of clinical benefits.

## 4. Materials and Methods

Study design and participants: This is a cross-sectional study containing census data from two hundred young (20–30 years old) non-obese (BMI: 18.5–29.9 kg/m^2^) healthy females retrieved from Qatar Biobank. Samples were selected based on gender (females), age (between 20 and 30), BMI (less than 30) and medical history (no chronic illnesses). Collected data included measurements of systolic and diastolic blood pressure, waist to hip ratio (WHR), BMI, clinical chemistry, endocrinology tests and metabolomics data. This study was approved by the Institutional Review Boards of the Qatar Biobank (Ex -2021-QF-QBB-RES-ACC-00007-0155) and Qatar University (QU-IRB 1493-E/21). The outcome variable is the binary BMI groups, as participants were classified according to their BMI into lean (BMI < 25, *n* = 108) and overweight (BMI ≥ 25, *n* = 92) groups. Validation of metabolic biomarkers was performed in 107 subjects (BMI < 25, *n* = 24, and BMI ≥ 25, *n* = 83, including 8 overweight and 75 obese). Characteristics of the validation cohort were previously published [9]. Protocols were approved by the Institutional Review Board of the Anti-Doping Laboratory Qatar (X2017000224) and Weill Cornell Medicine-Qatar (15-00007).

Metabolomics measurements: Established protocols were used for untargeted metabolomics of serum samples from all participants using the Metabolon platform [16]. Waters ACQUITY ultra-performance liquid chromatography (UPLC) and a Thermo Scientific Q-Exactive high-resolution/accurate mass spectrometer interfaced with a heated electrospray ionization (HESI-II) source and Orbitrap mass analyzer operated at 35,000 mass resolution were used for metabolite measurement. A detailed description of the methodology as previously described [16]. To identify compounds, hits were compared with already existing library entries of purified standards of over 3300 purified standard compounds. Compounds were then assigned to various categories according to their sources as previously described [17]. Although over 3300 metabolites were measured, we focused our study on 19 biomarkers of coronary artery disease published previously [15]. Out of the 19 biomarkers, only 17 were available in our database. Of the 17 available biomarkers, only five were statistically different between the BMI groups. The raw metabolomics data were previously published [12]. Among profiled metabolites, 17 metabolites previously shown to be associated with CHD risk were compared between the two studied groups, including nucleotides (uridine), carbohydrates (mannose), amino acids (dimethylglycine, asparagine, N-acetylalanine, indole-lactate, N-acetylthreonine, *p*-cresol sulfate, 2-methylbutyrylcarnitine, N-acetyl-1-methylhistidine), xenobiotics (theophylline, erythritol, 4-vinylphenol sulfate, O-sulfo-L-tyrosine) and lipids (linolenate alpha or gamma (18:3n3 or 6), 1-arachidonoyl-glycerylphosphorylcholine (GPC) (20:4n6), 13-hydroxyoctadecadienoic acid (HODE) and 9-HODE).

Statistical analysis: Multivariable logistic regression was used to identify a parsimonious model to explain the relationship between CHD metabolic biomarkers as continuous variables and overweight as a categorical variable. The model was repeated following the adjustment for creatinine as a potential confounder. The area under the receiver operating characteristic curve AUC and Hosmer and Lemeshow test was used to assess goodness of fit. Stepwise linear regression was performed to assess the correlation between five identified overweight-associated CHD metabolic predictors and classical risk factors using IBM SPSS version 25, R version 3.2.1. Multiple testing correction was performed, and the significance level was set accordingly. The analysis was carried out using IBM SPSS version 25 and STATA version 15. 5.

## 5. Conclusions

Our novel results provide evidence that apparently healthy overweight females exhibit significantly different metabolic biomarkers of CHD compared to their lean counterparts. Further validation of the potential use of the identified metabolites to predict increased CHD risk in certain apparently healthy overweight females is warranted.

## Figures and Tables

**Table 1:** General characteristics of participants. Abbreviations: BMI—body mass index, WHR—waist-to-hip ratio, SBP—systolic blood pressure, DBP—diastolic blood pressure, LDL—low-density lipoprotein, HDL—high-density lipoprotein, HbA1C—hemoglobin A1c, HOMA-IR—homeostatic model assessment of insulin resistance, ALP—alkaline phosphatase, ALT—alanine transaminase, AST—aspartate aminotransferase). Data are presented as mean (SD). Differences between lean and overweight were tested by independent sample t-test (normally distributed variables) or Mann–Whitney U (variables with skewed distribution) test. A *p*-value level of 0.001 was used to infer significance. Accordingly, significant differences are highlighted.

Measurement	Variables	Total	Lean	Overweight	*p*-Value
		(N = 200)	(N = 108)	(N = 92)		
Vital signs	Age (years)	29.1 (5.4)	28 (5.5)	30.4 (5.1)	0.073
	BMI (kg/m^2^)	24.7 (2.8)	22.5 (1.4)	27.3 (1.4)	0.000
	WHR	0.74 (0.1)	0.73 (0.1)	0.75 (0.1)	0.026
	SBP (mmHg)	101.1 (9.4)	99.9 (8)	102.5 (10.7)	0.047
	DBP (mmHg)	65.9 (8.1)	65.9 (6.9)	65.9 (9.3)	0.987
	Pulse (pulse per minute)	72.3 (9.3)	72.7 (9.6)	71.9 (8.9)	0.541
Lipid profile	Cholesterol total (mmol/L)	4.7 (0.7)	4.5 (0.7)	4.9 (0.8)	0.000
	HDL-cholesterol (mmol/L)	1.6 (0.3)	1.6 (0.3)	1.6 (0.3)	0.970
	LDL-cholesterol (mmol/L)	2.6 (0.7)	2.4 (0.6)	2.9 (0.6)	0.000
	Triglyceride (mmol/L)	1 (0.4)	0.9 (0.4)	1 (0.5)	0.042
Blood sugar	Fasting blood glucose (mmol/L)	4.8 (0.4)	4.7 (0.4)	4.8 (0.4)	0.030
	Insulin (μU/mL)	11.1 (8.3)	9.3 (6.5)	13.1 (9.7)	0.001
	HbA 1C (%)	5.2 (0.3)	5.1 (0.3)	5.2 (0.3)	0.055
	C-Peptide (ng/mL)	2.3 (1.1)	2 (1)	2.5 (1.3)	0.005
	HOMA-IR	2.4 (1.9)	2 (1.4)	2.9 (2.2)	0.001
Hormones	Thyroid stimulating hormone (mIU/L)	1.8 (1.4)	1.7 (1.2)	1.8 (1.6)	0.751
	Testosterone total (nmol/L)	µ1.2 (0.6)	1.2 (0.7)	1.2 (0.5)	0.942
	Estradiol (pmol/L)	518.8 (137.2)	608.7 (1821.9)	413.9 (451)	0.324
	Sex hormone-binding globulin (nmol/L)	92.7 (74.4)	13.6 (1.9)	13.4 (2.4)	0.195
	Free thyroxine (T4) (pmol/L)	13.5 (2.1)	13.6 (1.9)	13.4 (2.4)	0.620
	Free triiodothyronine (T3) (pmol/L)	4.5 (0.8)	4.4 (0.7)	4.4 (0.7)	0.335
Liver function	Bilirubin total (μmol/L)	6.2 (2.9)	6.1 (2.7)	6.2 (3.3)	0.829
	Albumin (g/L)	45.2 (2.3)	45.8 (2.5)	44.5 (1.9)	0.000
	Alkaline phosphatase (IU/L)	62.6 (20.5)	60.9 (15.7)	64.6 (24.9)	0.208
	ALT ( GPT ) (IU/L)	15 (10)	13.2 (6.8)	17 (12.5)	0.006
	AST (GOT) (IU/L)	16.8 (5.6)	16.1 (3.6)	17.6 (7.1)	0.055
Kidney function tests	Sodium (mmol/L)	139.8 (1.9)	139.8 (2)	139.7 (1.9)	0.775
	Potassium (mmol/L)	4.3 (0.3)	4.3 (0.4)	4.2 (0.3)	0.055
	Chloride (mmol/L)	101.5 (1.9)	101.6 (1.9)	101.5 (1.9)	0.698
	Bicarbonate (mmol/L)	25.7 (1.9)	25.7 (1.9)	25.7 (1.9)	0.992
	Urea (mmol/L)	3.9 (1.0)	3.9 (1)	3.9 (1)	0.929
	Creatinine (μmol/L)	55.6 (8.5	7.6 (54.3)	57.1 (9.2)	0.018
	Calcium (mmol/L)	2.37 (0.08)	2.38 (0.08)	2.36 (0.08)	0.047
	Calcium corrected (mmol/L)	2.3 (0.1)	2.3 (0.1)	2.3 (0.1)	0.990
	Phosphorus (mmol/L)	1.2 (0.2)	1.2 (0.2)	1.2 (0.2)	0.857
	Uric Acid (umol/L)	232 (50.8)	221 (44.1)	244.8 (55.2)	0.001
	Magnesium (mg/dL)	0.8 (0.1)	0.8 (0.1)	0.8 (0.1)	0.647
	Total protein (g/L)	73.5 (3.9)	64.2 (9.6)	65.5 (10.8)	0.105
	Homocysteine (μmol/L)	7.8 (2.6)	7.6 (2.6)	7.9 (2.7)	0.459
Ion profile	Iron (μmol/L)	13.3 (7.2)	14.1 (8.5)	12.3 (5.2)	0.079
	Total iron-binding capacity (mmol/L)	64.8 (10.2)	64.2 (9.6)	65.5 (10.8)	0.401
	Unsaturated iron-binding capacity (µmol/L)	51.6 (12.8)	50.2 (12.7)	53.1 (12.8)	0.111
	Ferritin (μg/L)	21.7 (23.3)	19.7 (17.6)	24 (28.5)	0.193
Vitamins	Folate (nmol/L)	24.7 (8)	25.3 (7.8)	24.1 (8.3)	0.294
	Vitamin B12 (nmol/L)	271 (108.7)	283.4 (119.2)	256.8 (94)	0.089
	Dihydroxyvitamin D Total (ng/mL)	17.1 (10.1)	16.2 (10.2)	18.1 (9.9)	0.198

**Table 2:** Coronary heart disease (CHD) classical traits and metabolites differentiating apparently healthy overweight from lean females. Logistic regression was used to assess the relationship between BMI groups and the CHD metabolites. OR—odds ratio, CI—confidence intervals. *p*-value significance levels of 0.003 and 0.01 were used in the discovery and replication cohorts, respectively, to infer significance.

Model	Variables	Beta	S.E.	*p* Value	OD (95% CI)	AUC (95% CI)
Classical risk factors	Glucose	0.8	0.4	0.035	2.1 (1.1−4.3)	0.77 (0.6−0.8)
	Creatinine	0.1	0.0	0.004	1.1 (1−1.1)	
	Albumin	−0.3	0.1	0.001	0.8 (0.7−0.9)	
Discovery cohort	1-Arachidonoyl-GPC (20:4n6)	2.1	0.6	0.001	8.1 (2.5−26.7)	0.76 (0.7−0.8)
	Asparagine	−2.4	0.8	0.002	0.1 (0−0.4)	
	Mannose	1.6	0.6	0.004	5.1 (1.7−15.5)	
	Linolenate (alpha or gamma; (18:3n3 or 6))	−0.7	0.2	0.002	0.5 (0.3−0.8)	
	Dimethylglycine	1.9	0.6	0.004	6.4 (1.8–22.4)	
Replication cohort	Asparagine	–1.6	0.7	0.019	0.2 (0.1–0.8)	0.97 (0.9–1)
	Mannose	1.7	0.7	0.017	5.3 (1.3–20.7)	
	Linolenate (alpha or gamma; (18:3n3 or 6))	–2.2	0.7	0.002	9.3 (2.3–37.6)	
	Dimethylglycine	–0.3	0.5	0.549	0.7 (0.3–2)	

**Table 3:** CHD metabolites in apparently healthy lean, and overweight females. Data are presented as mean (SD). Differences between lean and overweight were tested by an independent sample *t*-test. A *p*-value level of 0.01 was used to infer significance. Accordingly, significant differences are highlighted. Asterisks (*) indicated on IDs of some metabolites refer to compounds that have not been officially confirmed based on a standard, but their identities are known with confidence.

Variables	Total	Lean	Overweight	*p*-Value
	(N = 200)	(N = 108)	(N = 92)	
1-Arachidonoyl-GPC (20:4n6) *	0.94 (0.3)	0.89 (0.26)	1.01 (0.34)	0.001
Asparagine	1.06 (0.23)	1.09 (0.23)	1.03 (0.23)	0.005
Mannose	0.93 (0.29)	0.89 (0.29)	0.99 (0.28)	0.010
Linolenate (alpha or gamma; (18:3n3 or 6))	1.25 (0.82)	1.32 (0.81)	1.18 (0.83)	0.002
Dimethylglycine	0.93 (0.34)	0.89 (0.24)	0.99 (0.42)	0.006
Uridine	0.91 (0.27)	0.89 (0.27)	0.92 (0.27)	0.521
N-acetylalanine	0.95 (0.18)	0.92 (0.18)	0.98 (0.18)	0.043
13-HODE + 9-HODE	1.16 (0.53)	1.24 (0.52)	1.08 (0.53)	0.024
O-sulfo-L-tyrosine	0.94 (0.24)	0.91 (0.24)	0.97 (0.25)	0.377
4-Vinylphenol sulfate	1.14 (2)	1.14 (2.04)	1.13 (1.97)	0.376
N-acetylthreonine	0.98 (0.27)	0.96 (0.26)	1.01 (0.27)	0.192
N-acetyl-3-methylhistidine *	0.86 (0.42)	0.82 (0.29)	0.91 (0.51)	0.656
Theophylline	1.04 (0.75)	1.04 (0.84)	1.04 (0.64)	0.585
Erythritol	0.84 (0.59)	0.85 (0.75)	0.82 (0.3)	0.611
2-Methylbutyrylcarnitine (C5)	0.73 (0.37)	0.72 (0.4)	0.74 (0.34)	0.769
Indolelactate	0.81 (0.27)	0.8 (0.25)	0.82 (0.29)	0.246
p-Cresol sulfate	1.27 (0.83)	1.25 (0.76)	1.29 (0.91)	0.130

**Table 4:** Classical risk factors that best predict levels of CHD metabolites differentiating lean and overweight. A partial correlation analysis by stepwise linear regression was performed. Each model was run using 46 classical risk factors indicated in Table 1 per CHD metabolite. A *p*-value significance level of 0.001 was used for each model separately.

Metabolite	Predictors	Beta	*p* Value (Metabolite)	Adjusted R-Squared	*p* Value (Model)
Asparagine	Total cholesterol	−0.3	<0.001	0.15	0.00006
	Sex hormone-binding globulin	0.22	0.005		
	Calcium	0.22	0.006		
	Total testosterone	−0.2	0.022		
Mannose	Sex hormone-binding globulin	0.31	<0.001	0.15	0.00002
	Glucose	0.2	0.009		
	BMI	0.19	0.014		
	Total bilirubin	0.15	0.047		
1-Arachidonoyl-GPC (20:4n6)	Total cholesterol	0.85	<0.001	0.37	1.6 × 10^−12^
	Alkaline phosphatase	0.13	0.043		
	Homocysteine	−0.2	0.004		
	LDL-cholesterol	−0.5	<0.001		
	Total testosterone	−0.3	<0.001		
	Free thyroxine	0.19	0.004		
	Chloride	−0.1	0.045		
Linolenate (18:3n3 or 6)	C-Peptide	−0.4	<0.001	0.23	6.5 × 10^−9^
	DBP	0.19	0.008		
	Free triiodothyronine	0.18	0.014		
	Total iron-binding capacity	0.16	0.028		

## Data Availability

The datasets used and/or analyzed during the current study are available from the corresponding author on reasonable request. The data are not publicly available due to Qatar Biobank’s rules.
